# Gain‐of‐function p53 activates multiple signaling pathways to induce oncogenicity in lung cancer cells

**DOI:** 10.1002/1878-0261.12068

**Published:** 2017-05-08

**Authors:** Catherine A. Vaughan, Shilpa Singh, Steven R. Grossman, Brad Windle, Swati Palit Deb, Sumitra Deb

**Affiliations:** ^1^ Department of Biochemistry & Molecular Biology Virginia Commonwealth University Richmond VA USA; ^2^ Integrated Life Sciences Program Virginia Commonwealth University Richmond VA USA; ^3^ Massey Cancer Center Virginia Commonwealth University Richmond VA USA; ^4^ Department of Internal Medicine Division of Hematology Oncology and Palliative Care Richmond VA USA; ^5^ Philips Institute Virginia Commonwealth University Richmond VA USA

**Keywords:** activation, ChIP‐seq, gain‐of‐function, mutant, p53, transcription, transcription factor

## Abstract

Gain‐of‐function (GOF) mutants of p53 upregulate genes implicated in cell proliferation and oncogenesis. Here, we report that GOF p53 induces tumorigenicity through simultaneous activation of key oncogenic pathways including those controlling putative tumor‐initiating cell functions. We determined that in cells expressing p53‐R273H, GOF p53 simultaneously upregulates genes from multiple signaling pathways by recognizing promoters containing distinct transcription factor (TF) binding sites. Our analytical data support a model in which GOF p53 complexes with two TFs on the promoter—a mediator protein, Med17, and a histone acetyl transferase, activating histone acetylation—and enhances gene expression to signal cell proliferation and oncogenesis. Thus, therapeutic inhibition of one GOF p53‐induced pathway would be insufficient to prevent tumor growth as the oncoprotein activates a multitude of parallel pathways. This discovery suggests enormous selection advantage for cancer cells with GOF p53 to induce oncogenic growth, highlighting the problems of cancer therapy.

**Abbreviations**
ChIP: chromatin immunoprecipitation
EMT: epithelial‐to‐mesenchymal transition
GOF: gain of function
HAT: histone acetyltransferase
TAF: TATA box binding protein‐associated factor
TCGA: the cancer genome atlas
TF: transcription factor
TIC: tumor‐initiating cell

## 1 Introduction

p53 mutation is very common in human lung cancer: 33% in non‐small‐cell lung cancer to 70% in small‐cell lung cancer (Greenblatt *et al*., 1994). The majority of p53 mutations are found as single nucleotide changes resulting in missense mutations, representing gain‐of‐function (GOF) mutations having a ‘driver’ role in oncogenesis (Bronte *et al*., 2010; Vandin *et al*., 2012). The GOF activity of mutant p53 is observed as a number of different biological properties, including but not limited to increased tumorigenicity (Dittmer *et al*., 1993; Lanyi *et al*., 1998), metastasis and invasiveness (Taylor *et al*., 1992), growth rate (Murphy *et al*., 2000), genetic instability (Hanel and Moll, 2012), motility (Yeudall *et al*., 2012) and decreased sensitivity to chemotherapeutic drugs (Blandino *et al*., 1999; Scian *et al*., 2005). Mutant p53 GOF activity has also been demonstrated in mouse systems (Hanel *et al*., 2013; Liu *et al*., 2000; Olive *et al*., 2004). Work from our laboratory and others shows the feasibility of inhibition of GOF‐mutant p53 as a future cancer therapy (Vaughan *et al*., 2012a,b; Yan *et al*., 2008).

Using murine systems, it was reported that mutant p53 requires its transactivation property to induce GOF characteristics in cells (Lanyi *et al*., 1998; Lin *et al*., 1995). A number of GOF p53 target promoters and genes have been identified whose inhibition led to at least partial inhibition of GOF activities (Muller and Vousden, 2013); this led to the notion that GOF activities are at least partially conducted through the transactivation property of GOF p53 mutants (Freed‐Pastor and Prives, 2012; Muller and Vousden, 2013, 2014; Oren and Rotter, 2010). However, it is not known whether GOF p53 utilizes only one or more signaling pathways for its functions.

The mechanism of transactivation by GOF p53 can be explained by different forms of the following two nonmutually exclusive hypotheses: (a) GOF p53 interacts with p53 family members p73/p63 (or other cellular repressors), releasing their repression of gene expression leading to an apparent activation (Gaiddon *et al*., 2001; Liu *et al*., 2011; Melino, 2011; Santoro *et al*., 2014), and (b) GOF p53 directly upregulates expression of growth‐promoting and oncogenic genes. With the use of chromatin immunoprecipitation (ChIP), we and others have identified a number of promoters where GOF p53 interacts (Vaughan *et al*., 2012c, 2016); ChIP‐seq analysis was performed defining genes that are targets of GOF p53 that might explain different GOF activities of mutant p53 (Do *et al*., 2012; Stambolsky *et al*., 2010; Vaughan *et al*., 2014). However, the actual mechanism of transactivation by GOF p53 is unclear.

Here, ChIP and RNA‐seq analyses with human lung cancer cells expressing p53‐R273H revealed the sites on promoters/enhancers of genes where GOF p53 interacts and that are regulated by GOF p53. We show that specific transcription factors (TFs) are involved in mediating GOF p53 binding and transactivation of specific promoters. Thus, GOF p53 behaves as a pseudo‐opportunistic TF in selecting its partners for a gene that it induces. Functional relevance of different categories of genes that are upregulated by GOF p53 has also been evaluated by RNAi experiments. We established that GOF p53 induces oncogenesis by influencing TFs controlling a multitude of oncogenic genes and cancer signaling pathways including signaling pathways regulating tumor‐initiating cells (TICs), simultaneously. We suggest a model to explain GOF p53‐mediated gene induction and demonstrate that on the regulatory sequences of the gene that it activates, GOF p53 complexes with two TFs, a member of the mediator complex Med17, and a histone acetyl transferase activating histone acetylation fostering expression of oncogenic target genes.

## 2 Results

### 2.1 ChIP and RNA‐seq reveal sets of genes that are upregulated by p53‐R273H and whose regulatory sequences interact with mutant p53 in H1299 cells

To decipher the mechanism of transactivation by GOF p53, we first identified genomic promoter/enhancer sequences bound by GOF p53 by performing ChIP‐seq in cells overexpressing p53‐R273H. We then compared mutant p53 levels of different lung cancer cell lines used in the study. Figure S1A shows western analysis for mutant p53 levels in H1299 cells expressing p53‐R273H (or vector control) and lung cancer cells expressing endogenous p53 mutants: H1793, H1975, H2405, KNS‐62, and VMRC‐LCD. Figure S1B depicts a western blot after immunoprecipitations (IPs) under ChIP assay washing conditions for mutant p53 from different cell systems. Figure S1C shows plots comparing peaks from p53, AcH3, and IgG ChIP‐seqs using H1299 cells expressing p53‐R273H on chromatin; clear alignment of peaks can be observed.

Genes are selected so that the mutant p53 binding on their promoters are at least fivefold over the background with a significance of *P* < 0.003. Table 1 shows examples of genes that are upregulated by p53‐R273H (in RNA‐seq) and whose promoter/enhancers bind p53‐R273H. The genes are divided into different functional categories (DAVID annotation). The entire list of genes with GOF p53 bound to their promoter/enhancer is in Table S1.

**Table 1:** Identification of several genes considered to have p53 bound to the promoter in ChIP seq and that are up‐regulated in RNA seq *in vivo. P*‐value < 0.003 with an FDR < 0.02

Functional groups	Gene name		
Cell cycle components	APC	AURKB	CCNB1
	CCNB2	CCNE1	CCNA2
Subcellular components	CHK1	CTNNB1	MTG1
	NDUFA2	PC	
Cell proliferation	ABL1	AKT1	BAD
	BAX	TGFp	IGF1R
	PIM1		
Cell mobility	KIF2A	KIF2C	KIF3A
	TGFp		
Apoptosis and survival	ABL1	AVEN	BAD
	BAX	BCL10	Mcl1
Ser/Thr and other protein kinases	FLT4	AXL	CHK1
	CHK2	EGFR	MAPK1
	IGF1R	PIM1	
Carbohydrate metabolism	NDUFA2	NDUFA3	
Protein and nucleotide synthesis	AXL	DNMT3B	ENO1
	HES1	HEY1	
Transcription	AXL	CREB1	EIF3K
	NfkB2		
Replication	ANAPC4	CCNB1	CCNB2
Chromatin modification	DNMT3B	EZH2	HAT1
	HELLS		
Signal transduction for growth and oncogenesis	APC	BAX	BCL6
	CTNNB1		
TIC genes	ALDH1A1	DNMT3B	Nanog
	Oct4	Sox2	Notch

We next wanted to determine the genes whose levels of expression are modulated by GOF p53 via interaction of mutant p53 on the genes’ promoter/enhancer sequences and have performed RNA‐seq (identification of direct and indirect transcriptional targets of GOF p53). We have used the same H1299 cells expressing p53‐R273H that were used for mutant p53 ChIP‐seq to perform unbiased RNA‐seq. Table S2 shows the entire list of genes upregulated by p53‐R273H in comparison with control (vector). A complete list of genes common to RNA‐seq and p53 ChIP‐seq is in Table S3.

We also analyzed RNA‐seq data available in the cancer genome atlas (TCGA) to look for levels of expression of some of the genes that were found to be upregulated in our analysis with lung cancer cell lines. The method of data extraction from the TCGA database has been described in Materials and methods. Table S4 shows the extent of upregulation of expression of the genes as obtained from TCGA. For easy handling, we have combined samples with WT p53 and no p53 as non‐GOF p53.

### 2.2 Direct and indirect transcriptional targets of GOF p53

Analyses of RNA‐seq and p53 ChIP‐seq data led to the list of genes that are upregulated by mutant p53 and whose promoter/enhancers interact (or not) with mutant p53. The interaction of mutant p53 on promoter/enhancers and concomitant transactivation of genes by p53‐R273H suggest the possibility that mutant p53 indeed directly contributes to the transactivation and that these genes are direct targets of GOF p53. Table S5 shows examples of genes that do not have mutant p53 interactions on the regulatory sequences, but are upregulated by p53‐R273H (indirect transcriptional targets). A complete list of indirect targets is shown in Table S6. It is expected that for indirect targets (among a few possibilities), mutant p53 either releases some inhibition of expression or activates another transactivator.

### 2.3 Direct target genes interact with GOF p53 on their promoter/enhancers and get induced

**Figure 1:**
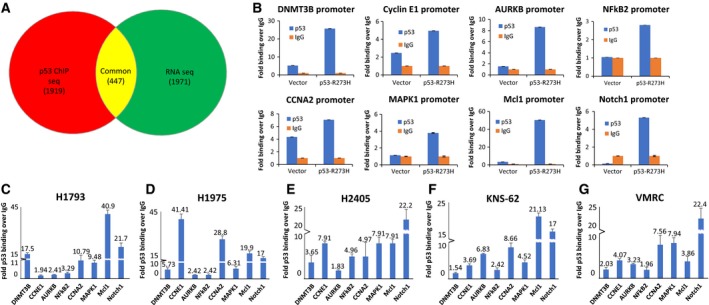
Identification of genes that are induced by p53‐R273H and directly interact with GOF p53. Unbiased RNA‐seq analyses have been performed using RNA from H1299 cells expressing p53‐R273H (or vector control) that were also used for mutant p53 ChIP‐seq to determine what genes are modulated by GOF p53. Details of the experiment have been described in Methods. (A) Sequence reads from RNA and ChIP‐seq data sets have been analyzed using the ArrayStar program (DNASTAR). Genes that were upregulated at least threefold over control in the RNA‐seq and that had a greater than fivefold increase in mutant p53 binding in ChIP‐seq were compared. A Venn diagram showing the overlap of RNA and ChIP‐seq gene lists is shown. There were 447 genes in common from the two data sets. (B) Verification of the p53 ChIP‐seq data for eight target genes shown in Table 1 was performed using independent ChIP followed by QPCR. ChIP was performed in triplicate, and qPCR was performed for each sample in duplicate. We have also tested whether regulatory sequences of genes that were identified by ChIP‐seq analysis of H1299 cells expressing p53‐R273H bind mutant p53 in lung cancer cell lines with endogenous GOF p53 expression: (C)‐H1793 (p53‐R273H), (D)‐H1975 (p53‐R273H), (E)‐H2405 (p53‐R273H), (F)‐KNS‐62 (p53‐R249S), and (G)‐VMRC‐LCD (p53‐R175H). p53 ChIP assays were performed as described in the text followed by QPCR analysis. Data are plotted as fold p53 binding over IgG control after normalization with input DNA. Individual fold values are indicated above the bars for C–G. ChIP was performed in duplicate, and qPCR was performed for each sample in duplicate. All error bars were calculated using standard deviation. We show that the endogenous GOF p53 in our lung cancer cell lines interacts with the eight direct target genes to varying degrees, but regardless of the cell line.

To get an idea about how many genes that are bound to GOF p53 are getting activated and vice versa, we have performed a simple bioinformatic approach. Figure 1A displays a Venn diagram showing overlap of RNA and ChIP‐seq gene lists. At least 18% of upregulated genes are direct targets, with GOF p53 interacting on the promoter/enhancers. We validated the ChIP‐seq data by independent p53 ChIP (Fig. 1B). We also tested whether regulatory sequences of genes identified by ChIP‐seq analysis bind to mutant p53 in lung cancer cell lines with endogenous GOF p53; p53 ChIP verified our expectation (Fig. 1C–G). The commonality of ChIP‐seq data and independent ChIP analyses performed with cell lines containing endogenous GOF p53 indicate that these GOF p53s must be interacting with these promoter/enhancers in a mechanistically similar way. We have also performed quantitative PCR (qPCR)( on control regions of DNA adjacent to where p53 interacts but where our ChIP‐seq data showed no interactions for the targets shown in Fig. 1B. Figure S2A shows no p53 interaction at these sites. Three lung cancer cell lines were used to validate CDH2 and MAPK8, two indirect targets of GOF p53, by ChIP, which showed insignificant GOF p53 interactions on promoter/enhancer sequences (Fig. S3).

**Figure 2:**
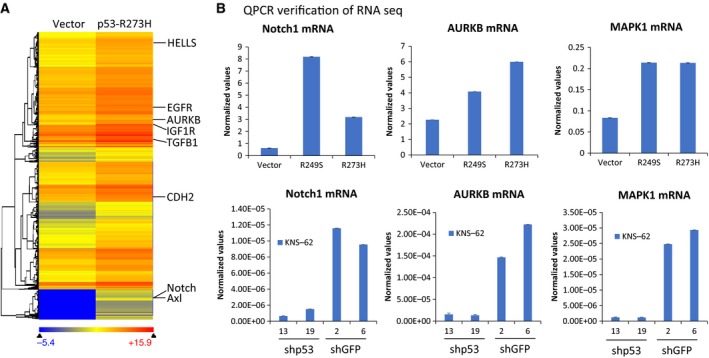
Analysis of RNA‐seq data. (A) Individual RNA‐seq fastq files from H1299 cells stably expressing p53‐R273H or vector control were analyzed using the ArrayStar program (DNASTAR). Hierarchical clustering of genes that had a greater than fivefold increase in R273H versus vector control was performed and is shown. We note that there are clusters of genes that seem to be turned on by GOF p53, for example, Axl and Notch‐1 (group at the bottom), while other genes are already expressed in the control cells but expression is greatly increased in the presence of GOF p53. (B) Verification of three genes, Notch1, AURKB, and MAPK1, from the RNA‐seq data by RT‐QPCR analysis using RNA from H1299 cells expressing different GOF p53s as well as the lung cancer cell line KNS‐62 endogenously expressing p53‐R249S and its p53 knockdowns. Numbers along the *x*‐axis indicate different clones of shp53 and shGFP. Two independent preparations of RNA from both H1299 and KNS‐62 sets of cells were prepared. qPCR was performed in duplicate, and values were normalized with GAPDH. Error bars were calculated using standard deviation.

We have attempted to determine whether we can group some of the genes that are activated by GOF p53 and performed cluster analysis of RNA‐seq. Figure 2A shows analysis of RNA‐seq data from vector and p53‐R273H‐transfected (stable) H1299 cells depicting a range of genes that are upregulated by mutant p53. Genes corresponding to the blue cluster in the vector lane represent a group of genes, which includes Notch and Axl, with no or little expression that is strikingly turned on by GOF p53. In contrast, the other clusters of genes that include IGF1R and TGFβ are upregulated from a basal level. Figure 2B shows independent RT‐QPCR verification of expression levels of three genes, Notch1, AURKB, and MAPK1, from Table 1; also shown are RT‐QPCR data looking at expression levels of these genes in the lung cancer cell line KNS‐62 and its p53 knockdown. Data from our knockdown cell systems corroborated observations from H1299 cell systems, confirming that these genes are upregulated by GOF p53. We show upregulation of three additional genes, CCNE1, NFkB2, and cyclin A2 (CCNA2), by RT‐QPCR in H1299 cells expressing p53‐R273H versus a vector control (Fig. S4A). Protein upregulation is also seen for CCNA2, CCNE1, and MAPK1 (Fig. S4B).

### 2.4 GOF p53 upregulates genes related to TICs

As we noticed GOF p53 upregulated many growth‐promoting genes, we wanted to test how GOF p53 regulates TIC‐related genes. Expression of a number of TIC‐related genes (Gerhardt *et al*., 2014; Giovannini *et al*., 2014; Iv Santaliz‐Ruiz *et al*., 2014; Li *et al*., 2014; Murakami *et al*., 2014; Qian *et al*., 2015; Singh *et al*., 2013; Sullivan *et al*., 2010) including Notch 1‐4, DNMT3B, Mcl1, aurora kinase B (AURKB), Oct4, Sox2, and Nanog are upregulated by p53‐R273H (2‐ to 12‐fold) and were found to have GOF p53 interact on their promoter/enhancers (Fig. S5A). Figure S5B also shows that one Notch ligand, JAG1, is also upregulated by p53‐R273H, suggesting the possibility that GOF p53 activates the Notch pathway. Thus, GOF p53 may regulate the pathway controlling growth of TIC.

### 2.5 Multiple oncogenic pathways are utilized for p53 GOF activities

Next, we wanted to get a picture of cellular pathways affected by GOF p53‐mediated transactivation. We tested the functional importance of a few genes (Table 1) representing different oncogenic pathways using three GOF assays: growth, migration, and invasion. The results of these assays are tabulated in Table S7 and the assays are shown in Fig. S6. GOF activities are affected by different genes participating in different functionally important signaling pathways, examples of which are shown in Fig. S7. Our work showed that genes important in the cell cycle, in apoptosis, transcription, and survival for TICs, and several signaling pathways including the EGFR, IGF1R, TGFβ, NFκB, and Notch pathways play significant roles in GOF activities.

### 2.6 GOF p53 regulates the Notch signaling pathway

**Figure 3:**
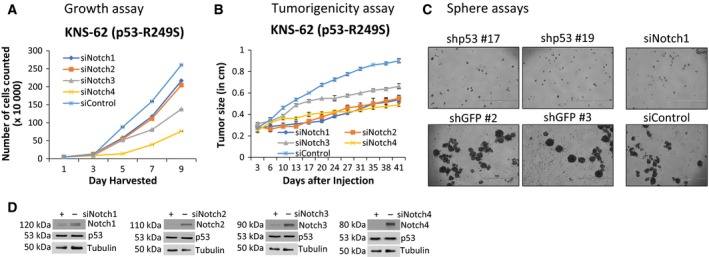
Notch pathway is involved in p53 GOF activity. We used siRNA against the four Notch genes to determine whether depletion of Notch would impact GOF activities of mutant p53 using KNS‐62 (p53‐R249S) cells. (A) Cell growth assays were carried out in triplicate as described in Methods. The results demonstrate that reduction in the four Notch genes reduces proliferation. (B) Tumorigenicity assay was carried out using xenografts in immunocompromised mice as described in Methods. Xenograft assay of siNotch‐depleted KNS‐62 cells was performed in duplicate and shows a reduction in tumorigenicity in nude mice. Both types of GOF activities are affected by Notch reduction. We note that depletion of different Notch proteins has different biological effects, although it is possible that some of it is related to the extent of knockdown. (C) Sphere assays were performed in triplicate on H1975 cells with stable p53 knockdown (or GFP control) and on H1975 cells after siRNA knockdown of Notch1, showing that the Notch pathway plays an important role in GOF activity of mutant p53 including the TIC functions. (D) Western blot analysis shows efficiency of siRNA knockdowns used in A–C.

We then tested whether Notch is required for GOF activities of mutant p53, and used RNAi to lower Notch levels in lung cancer cells KNS‐62. Figure 3A,B shows cell growth and tumorigenicity assays using KNS‐62 cells when we use siRNA against Notch. The data show that Notch knockdown causes a remarkable reduction in tumor and cell growth even though mutant p53 levels remain stable (Fig. 3D).

As the Notch pathway is involved in TIC functions, and targeting Notch may inhibit TICs, we tested spheroid formation after knockdown of Notch and compared that with cells that were depleted of GOF p53 to see whether there was any similarity. Spheroid formation is a widely used assay to study the TIC subpopulation within a group of cells (Cao *et al*., 2011). Figure 3C shows that RNAi treatment of H1975 cells with shRNA against p53 and siRNA against Notch both cause reduction in the number of spheroids formed. Thus, activation of the Notch pathway by GOF p53 plays an important role in GOF‐mutant p53 regulation of TIC functions.

It should be noted that by siRNA treatment against a gene, no change in p53 level has occurred (Fig. 3D). Therefore, GOF p53 induces simultaneous expression of different oncogenic and growth‐promoting genes for its GOF property, and reduction in any of the gene products has deleterious consequences on tumorigenicity.

### 2.7 p53‐R273H induces changes in AcH3 histone binding on the genome

**Figure 4:**
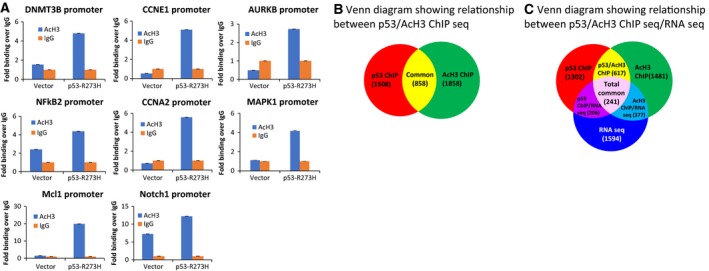
Direct targets of p53‐R273H that have increased H3 histone acetylation on the gene regulatory sequences. (A) Examples of genes shown in Table S9 representing direct targets of GOF p53 presumably activated through acetylation of histone H3 are shown here by QPCR verification. Independent ChIP against acetylated histone H3 was performed using H1299 cells stably expressing p53‐R273H or vector control. Data are plotted after normalization with input DNA. The methods used have been discussed in the text. ChIP was performed in duplicate, and qPCR was performed for each sample in duplicate. All error bars were calculated using standard deviation. (B) Sequence reads from p53 and AcH3 ChIP‐seq data sets were analyzed using the ArrayStar program (DNASTAR). Genes that had a greater than fivefold increase in p53 or AcH3 binding to the regulatory sequences of target genes in ChIP‐seq were compared. A Venn diagram showing the relationship between p53/AcH3 ChIP‐seq is shown with 858 genes in common between the two data sets. (C) Sequence reads from RNA, p53, and AcH3 ChIP‐seq data sets were analyzed using the ArrayStar program (DNASTAR). Genes that had a greater than threefold increase in upregulation by GOF p53 and that had a greater than fivefold increase in p53 or AcH3 binding to the regulatory sequences of target genes in ChIP‐seq were compared. A Venn diagram showing the relationship between p53/AcH3 ChIP‐seq and RNA‐seq is shown with 241 genes in common between the three data sets.

Once we had established some major pathways through which GOF p53 may act for its oncogenic activities, we started deciphering the molecular details of mechanism of transactivation by GOF p53. One probable mechanism for GOF p53‐induced transactivation is through the induction of histone acetylation, making the promoters more transcriptionally active. We have performed AcH3 histone ChIP‐seq with H1299 cells expressing p53‐R273H (or vector control). Table S8 shows the complete list of genes found to have increased binding of AcH3 histone. Table S9 shows examples of direct targets of p53‐R273H, which have increased AcH3 histone on chromatin on their regulatory sequences. Figure 4 depicts QPCR verifications from independent AcH3 ChIP. Table S10 lists genes that are in common between p53 and AcH3 ChIP‐seqs. Figure 4B shows a Venn diagram depicting the overlap of genes bound by GOF p53 and AcH3 histone. Genes in common between p53 and AcH3 ChIP‐seq data sets represent 36.26% of the p53 ChIP‐seq list and 31.59% of the AcH3 ChIP‐seq list. In both sets of data, we took genes that had at least an average of fivefold increase in binding in the presence of mutant p53 and whose *P*‐values were < 0.001. Figure 4C shows the commonality between genes that bind GOF p53, have induced histone acetylation, and enhanced gene expression. Genes in common between the p53 ChIP‐seq, AcH3 ChIP‐seq, and RNA‐seq data sets represent 10.18% of the p53 ChIP‐seq list, 8.87% of the AcH3 ChIP‐seq list, and 9.96% of the RNA‐seq data set. We propose that for a significant number of direct targets of GOF p53, histones get acetylated because of mutant p53's interaction on the promoter/enhancers with histone acetyl transferases (HATs); this leads to increased access of TFs to regulatory sequences for enhanced transcription (see Fig. 8).

### 2.8 GOF p53 nucleates on the target gene promoter/enhancer through its interaction with one or more TFs

**Figure 5:**
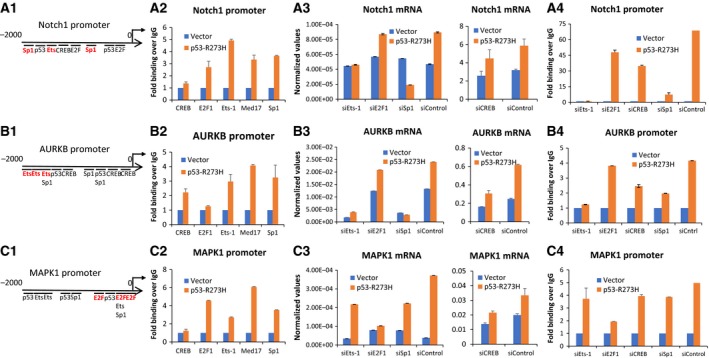
Specific TFs are needed for GOF p53‐mediated transactivation of target genes. A. (A1) Notch1 promoter schematic indicating different TF binding sites. (A2) TF ChIP analysis on the Notch1 promoter as analyzed by ChIP for individual TFs followed by QPCR. Data were normalized using input DNA and fold p53 binding over IgG was plotted. (A3) Notch1 mRNA expression analysis showing the effect of H1299 cells expressing p53‐R273H (or vector alone) transfected with siRNA against TFs on the ability of mutant p53 to transactivate the gene. Data represent QPCR values normalized to GAPDH which is not affected by mutant p53 (for mRNA expression). (A4) H1299 cells expressing p53‐R273H (or vector alone) were transfected with RNAi against TFs that had binding sites within the promoter (or control siRNA) and p53 ChIP was performed. Data were normalized using input DNA and fold p53 binding over IgG was plotted. The analysis performed in (A) above was performed for the AURKB promoter (B) as well as the MAPK1 promoter (C). The data shown indicate interaction of mutant p53 with Sp1 and Ets‐1 while bound on the Notch1 promoter region, with Ets1, Sp1, and somewhat CREB for the AURKB promoter, and with E2F1 on the MAPK1 promoter. QPCR analysis was performed as described in Methods using gene‐specific primers. Parallel western blot analysis to verify efficient knockdown of TFs studied is shown in Fig. S9A. Experiments were performed in triplicate. Error bars showing standard deviations are indicated.

As GOF p53 has not been shown to bind DNA sequence specifically, we surmised that GOF p53 does not bind DNA directly. We investigated whether GOF p53 requires interaction with TFs that bind DNA within promoter/enhancers. We selected three genes: Notch1, AURKB, and MAPK1, which have different TF requirements for their activity (Cordoba *et al*., 2016; Tessari *et al*., 2003; Yang *et al*., 2003). We used RNAi against several TFs in H1299 cells expressing p53‐R273H, and performed TF ChIP, gene expression analysis, and p53 ChIP to determine any possible interactions between TFs and GOF p53 on promoter/enhancers. Figure S8 shows the location of acetylated histone H3, p53, and IgG control peaks on genomic DNA where the Notch1 (S8A), AURKB (S8B), and MAPK1 (S8C) genes are located. The three genes regulating different pathways are shown in Fig. 5: Notch1 (Fig. 5A), AURKB (Fig. 5B), and MAPK1 (Fig. 5C). TF binding sites are indicated for the Notch1 (5A1), AURKB (5B1), and MAPK1 (5C1) promoters. While Notch1 has been reported to require Sp1 for its activation, AURKB has been published to require Ets and MAPK1 was published to require E2F1 (Cordoba *et al*., 2016; Wakahara *et al*., 2008; Yang *et al*., 2003). To show the requirement of different TFs for the three promoters, we first performed ChIP using antibodies against indicated TFs as well as Med17, a member of the mediator complex, because it is known to interact with p53 (Meyer *et al*., 2010). Figure 5A2,B2,C2 illustrates that the TFs bind to the three promoters. siRNA was used to knockdown expression of Ets‐1, Sp1, E2F1, and CREB, and western analysis demonstrating respective siRNA knockdown of the TFs is shown in Fig. S9A. Interestingly, when we knockdown expression of Ets‐1 and Sp1 (but not E2F1 or CREB), we lose the transactivation of Notch1 (5A3). On the other hand, when we knockdown expression of Ets‐1, Sp1, and CREB to an extent (but not E2F1), we lose the transactivation of AURKB (5B3). Knockdown of E2F1 and CREB to an extent (but not Ets‐1 or Sp1) reduced the level of MAPK1 mRNA (5C3). We have also knocked down expression of the four TFs and performed p53 ChIP to determine whether the TFs are required for increased mutant p53 binding to the three promoters. Figure 5A4 shows that when we knockdown Ets‐1 and Sp1 (but not E2F1 or CREB), we see reduced recruitment of mutant p53 to the Notch1 promoter, which corroborates our mRNA data in Fig. 5A3. Alternatively, Ets‐1, Sp1, and CREB knockdown inhibits p53 binding to the AURKB promoter with siEts‐1 showing the strongest effect (Fig. 5B4), and only E2F1 knockdown reduces p53 binding to the MAPK1 promoter (Fig. 5C4), while the other TF knockdowns did not affect p53 interacting with those promoters. Table S11 lists which TF is required for each gene in the three assays. We also show that there is no interaction of the respective TFs on nonspecific regions of DNA near the three gene promoters. Figure S9B demonstrates this fact. Figure S9C also shows that there is no interaction between p53 and the control regions of DNA and that this is irrespective of whether the Ets‐1, E2F1, CREB, or Sp1 TFs are present or not. Data shown in Fig. 5 demonstrate that Notch activation by GOF p53 requires two TFs, while the other two promoters seem to need one TF each. The genomic interaction of mutant p53 seems to be dependent on its interaction with TFs and seems to require more than one TF binding site (Fig. 8), in agreement with our previously published data (Vaughan *et al*., 2016).

**Figure 6:**
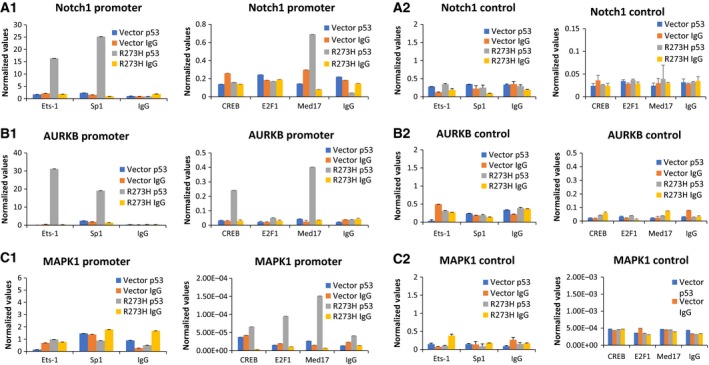
GOF p53 interacts with different TFs on the promoters of target genes. Detailed methods of ChIP‐re‐ChIP assays are given in Methods. H1299 cells expressing p53‐R273H (or stably transfected with vector control) were used. Antibodies against p53 were used for the first ChIP immunoprecipitation, and then specific antibodies for various TFs were used for the second ChIP immunoprecipitation. Promoter‐specific primers have been used in QPCR to detect three direct target promoters. (A) ChIP‐re‐ChIP of H1299 cells expressing p53‐R273H (or vector alone) showing different p53/TF interactions on the Notch1 promoter. GOF p53 interacts with Ets‐1, Sp1, and Med17. (A2) qPCR was performed on ChIP DNA from A1 using primers adjacent to where p53 was seen to interact on the promoter but where there was no interaction itself. Data show that p53 does not interact with the control regions of DNA. (B) ChIP‐re‐ChIP of H1299 cells expressing p53‐R273H (or vector alone) showing different p53/TF interactions on the AURKB promoter. GOF p53 interacts with Ets‐1, Sp1, CREB, and Med17. (B2) qPCR was performed on ChIP DNA from B1 using primers adjacent to where p53 was seen to interact on the promoter but where there was no interaction itself. Data show that p53 does not interact with the control regions of DNA. (C) ChIP‐re‐ChIP of H1299 cells expressing p53‐R273H (or vector alone) showing different p53/TF interactions on the MAPK1 promoter. GOF p53 interacts with E2F1 and Med17. (C2) qPCR was performed on ChIP DNA from C1 using primers adjacent to where p53 was seen to interact on the promoter but where there was no interaction itself. Data show that p53 does not interact with the control regions of DNA. Antibodies used for the first IP are indicated in the body of the figure, and antibodies used for the second IP are shown on the *x*‐axis. Data were normalized to input DNA. Experiments were performed in triplicate. Error bars showing standard deviations are indicated.

To investigate the specific GOF p53/TF interactions on the Notch1, AURKB, and MAPK1 promoters, we performed ChIP‐re‐ChIP analysis (Fig. 6). Antibodies against p53 were used for the first immunoprecipitation (IP), and antibodies recognizing different TFs were used for the second IP. Thus, for our ChIP‐re‐ChIP studies, the interactions between GOF p53‐Ets‐1, GOF p53‐E2F1, GOF p53‐CREB, and GOF p53‐Sp1 on the Notch1, AURKB, and MAPK1 promoters were studied. We saw that GOF p53 interacts with both Ets‐1 and Sp1 on the Notch1 promoter, while our analysis showed no interaction between mutant p53 and either CREB or E2F1 (Fig. 6A1). On the other hand, in the case of AURKB and MAPK1, which indicated the necessity of Ets1 and Sp1 for AURKB or E2F1 for MAPK1 in Fig. 5B4,C4 for p53 binding to the promoters, our ChIP‐re‐ChIP showed strong interactions between those TFs and GOF p53, while no interaction between mutant p53 and either Ets‐1 or Sp1 was seen for MAPK1 (Fig. 6B1,C1). Although our analysis indicates that GOF p53 interacts with different TFs depending on the nature of the gene (Figs 5 and 6), we noticed that in all three cases it interacts with MED17, suggesting a commonality between the three promoters (Fig. 6A1,B1,C1), and perhaps raises the possibility that Med17 may play an important role both structurally and functionally. We performed QPCR using the ChIP‐re‐ChIP DNA to see whether there was any interaction between p53 and the different TFs on the control region of DNA near the three gene promoters. Figure 6A2,B2,C2 shows that there is no binding at those locations.

**Figure 7:**
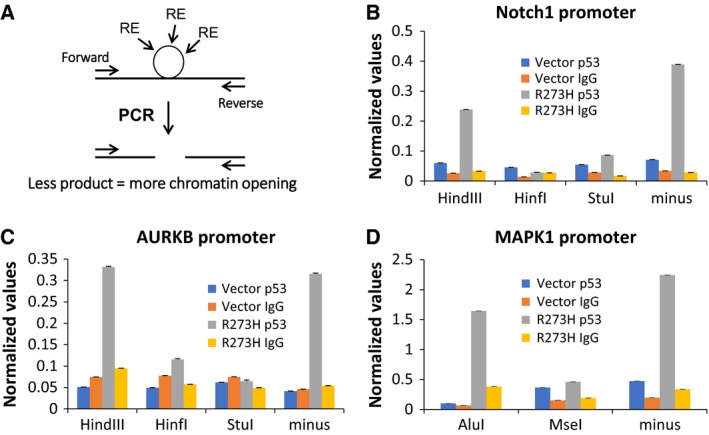
Proposed model for GOF p53 binding to induce chromatin opening. (A) Model showing the theory of our loop ChIP approach. ChIP DNA is digested with restriction enzymes that have sites located within the sequence that is amplified using ChIP primers. After digestion and PCR, less or no product that was formed indicated more chromatin opening. ChIP was performed on H1299 cells expressing p53‐R273H (or transfected with empty vector) and digested with enzymes indicated on the *x*‐axis. (B) Chromatin opening of the Notch1 promoter that contains HinfI and StuI sites, while HindIII was used as a negative control. (C) Chromatin opening of the AURKB promoter that contains HinfI and StuI sites, while HindIII was used as a negative control. (D) Chromatin opening of the MAPK1 promoter that contains a MseI site, while AluI was used as a negative control.

Next, we wanted to test whether we can detect chromatin opening caused by the complex assembled by GOF p53 and TFs, and determined whether the Notch1, AURKB, and MAPK1 promoters are in the ‘open’ state for transcription using a ‘chromatin loop assay’. A schematic of chromatin looping is shown in Fig. 7A. Using ChIP DNA, we cut the chromatin with restriction enzymes that have sites located within the primers we used for PCR amplification. If the DNA ‘loop’ is in an ‘open’ state, it will be cut with the enzyme and either significantly less or no PCR product will be formed. Figure 7B shows that the Notch1 promoter is susceptible to digestion by HinfI and StuI, but not by HindIII that does not have a site and was used as a control, to indicate that Notch1 is in the open state. The AURKB promoter (Fig. 7C) was successfully cut with HinfI and StuI, but not by HindIII (control). Similarly, the MAPK1 promoter (Fig. 7D) was cut by MseI, but not by AluI (control). These data indicate that GOF p53 induces chromatin opening.

## 3 Discussion

**Figure 8:**
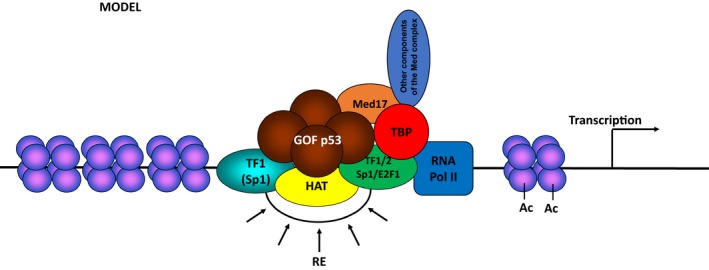
Proposed model for GOF p53‐induced promoter activation. Arrow toward the right‐hand side depicts transcription direction. The model proposes a single tetramer of GOF p53 bridges to interact with multiple TFs resulting in nucleation of HAT along with the mediator complex, Med17, in particular, and RNA Pol II. We also show a DNA loop formed in which restriction enzyme sites are located. It is assumed that GOF p53 induces histone acetylation through the action of HAT such as p300/CBP with which it interacts. For simplicity, TAF and other factors are not shown. With respect to mutant p53, it is not known at what point this complex formation happens, whether GOF p53 binds to different TFs on the regulatory sequences of target genes and then brings in HAT and the mediator complex to initiate transcription, or whether the complex forms in the nucleoplasm and then interacts with the DNA.

In this communication, we report that in lung cancer cells, many of the genes whose promoter/enhancers interact with GOF p53 are upregulated by mutant p53 (Table 1); a significant number of these gene promoter/enhancers also have increased acetylation of bound histone H3 (Table S9). Increased histone acetylation on chromatin indicates enhanced chromatin opening with easier TFs access to the DNA presumably for increased transcription rate (Fig. 8).

Our data with siRNA against TFs indicate that different TFs control transactivation of GOF p53 as well as GOF p53's ability to interact (perhaps not directly) with the promoter/enhancer depending on the gene. This suggests that GOF p53 uses different TFs to interact with promoter/enhancer sequences as not all promoters were seen to be affected the same way with siRNA against one TF or in TF ChIP and ChIP‐re‐ChIP (Figs 5 and 6). Interestingly, Med17, which is involved in transcription of genes (Kikuchi *et al*., 2015; Meyer *et al*., 2010), has been found to interact with all the promoter/enhancers tested as well as in all ChIP‐re‐ChIP assays (Figs 5 and 6). siRNA against Med17 causes a reduction in p53 binding to several GOF p53 target promoters (data not shown). So, the eventual model must account for all these facts. We can speculate that GOF p53 has a certain amount of promiscuity in interacting with TFs and gets nucleated on a promoter once it finds a suitable partner and then makes further contacts with Med17 and histone acetyltransferase (HAT) such as CBP/p300 to induce chromatin opening and increases the transcriptional rate. Whether Med17 is required for the complex formation and transactivation by GOF p53 is an important question whose answer is unknown at this time. We propose a new model (Fig. 8) to explain GOF p53‐mediated transactivation and show GOF p53 forms complexes with two TFs (perhaps not directly) on the promoter, whether they are the same or different, in agreement with our previously published data (Vaughan *et al*., 2016). It is, however, not clear yet whether any part of the GOF p53/TF/HAT/mediator complex forms first and is then nucleated on the DNA.

We have used RNAi technology to test whether different signaling pathways implicated by DAVID analysis have functional relevance, and concluded that GOF activities are regulated by simultaneous activation of multiple parallel as well as overlapping pathways to induce oncogenic progression (Table 1, Figs [Link], [Link], and Table S7). By reducing expression of one protein, GOF p53 may still induce tumorigenicity via activation of the other pathways. This discovery suggests enormous selection pressure on cancer cells to produce oncogenic growth; it also highlights the problems faced in treating cancer cells as their oncogenicity is fed by a multitude of parallel pathways.

We discovered that GOF p53 induces Notch expression activating the Notch pathway (Fig. 3 and Fig. S5). GOF p53 through the Notch pathway may regulate a series of oncogenic events involved in cancer progression, such as motility, invasion, growth, metastasis, epithelial‐to‐mesenchymal transition (EMT), and angiogenesis. Activation of the Notch pathway is related to TIC functions (Alamgeer *et al*., 2013; Hassan *et al*., 2013) and has been observed in various cancers including lung cancers (Wael *et al*., 2014; Yen *et al*., 2015). We demonstrate that GOF p53 activates Notch and its ligand JAG1 (Fig. 3 and Fig. S5) in lung cancer cells. In addition, we have seen a moderate upregulation of several Notch target genes, HES1 and HEY1, by GOF p53 (not shown) This upregulation confirms earlier reports of GOF p53 modulating EMT (Kogan‐Sakin *et al*., 2011). Thus, it appears that Notch pathway activation plays an important role in the GOF p53 path of oncogenesis.

The current work dealt with an unsolved aspect of GOF p53 biology, how GOF p53 affects different oncogenic processes using different signaling pathways and activates transcriptional roadways interacting with several TFs rather promiscuously getting nucleated on gene promoters. It uncovers a list of critical genes that may be targeted for cancer therapeutic purposes.

## 4 Materials and methods

### 4.1 Cells

Lung adenocarcinoma (LUAD) cell lines H1299 (p53‐null), VMRC‐LCD (p53‐R175H), H1975 (p53‐R273H), H2405 (p53‐R273H), and H1793 (p53‐R273H) and squamous cell carcinoma cell line KNS‐62 (p53‐R249S) were all purchased from commercial sources and were maintained in media as suggested by the suppliers. Methods for lipofection and nucleofection and generation of stable transfectants were as described earlier (Frum *et al*., 2009; Vaughan *et al*., 2012a,c). p53 knockdown (or shGFP control) clones were isolated using puromycin selection at 1 μg·mL^−1^.

### 4.2 H1299 cells expressing GOF p53 mutants

We have used H1299 cells expressing p53‐R273H and ‐R249S (or vector‐transfected) as described earlier (Scian *et al*., 2005).

### 4.3 siRNA transfection

siRNAs were nucleofected as indicated following the manufacturer's instructions (Lonza, Walkersville, MD, USA). Occasionally, transfections of siRNAs were performed by Lipofectamine 2000 (Thermo Fisher Scientific, Waltham, MA, USA). siRNA sequences used were as follows: siNotch1 5′‐GATGCGAGATCGACGTCAA‐3′, siNotch2 5′‐GGAGATGACTGCAGTGAGA‐3′, siNotch3 5′‐TCAATGCTGTGGATGAGCTTGGGAA‐3′, siNotch4 5′‐GGTTTCATAGGCCCAGACTGT‐3′, siTGFβ 5′‐GGACTATCCACCTGCAAGACT‐3′, siMcl1 5′‐CCCGCCGAATTCATTAATTTA‐3′, siPim1 5′‐CCATGGAAGTGGTCCTGCTGAAGAA‐3′, siCCNB2 5′‐GAGAATCTCTGCCAAGCTT‐3′, siHELLS 5′‐TAATGATGCTACTTCGTAA‐3′, siNFκB2 5′‐GCCCTGAGTGCCTGGATCT‐3′, siIGF1R 5′‐ATACGGATCACAAGTTGAG‐3′, siAxl 5′‐GAGATGTGACACATGACATG‐3′, siEts‐1 5′‐ACTTGCTACCATCCCGTAC‐3′, siE2F1 5′‐ATGCTACGAAGGTCCTGACACGTCA‐3′, siSp1 5′‐GGTAGCTCTAAGTTTTGAT‐3′, siCREB 5′‐AATACAGCTGGCTAACAATGG‐3′, and siControl 5′‐TCTTAATCGCGTATAAGGC‐3′.

### 4.4 Growth assays

Growth assays were performed as described by us with slight modifications (Scian *et al*., 2004). Cells were plated at a density of 50 000 cells/6‐cm dish in triplicate for five time points, trypsinized, and counted using a Coulter Counter (Beckman, Indianapolis, IN, USA). For siRNA treatment of cells, siRNA transfection was carried out for two consecutive days before starting the growth assay. Graphs represent the average of triplicate values for each experiment. Error bars shown represent standard deviations.

### 4.5 Cell migration assays

Cell migration was determined by wound closure assays described previously (Vaughan *et al*., 2016). Briefly, cells were trypsinized, counted, plated in both chambers of tissue culture inserts (ibidi USA, Inc., Fitchburg, WI, USA) in triplicate, and then grown to confluence. The insert was removed, and the distance across the cell‐free zone was measured (axiovision software; Carl Zeiss Microimaging, Thornwood, NY, USA). Cultures were returned to the incubator were allowed to migrate for 8 h, and the width of the cell‐free zone was re‐measured. Graphs represent the average of triplicate values for each experiment. Error bars shown represent standard deviations.

### 4.6 Invasion assays

Invasion assays were carried out as described (Wang *et al*., 2009). Matrigel was diluted in serum‐free media and aliquots were used to coat Transwell chambers. Cells were counted and seeded on top of the matrix in triplicate. Media were added to the lower chamber and plates were incubated at 37 °C for 24 h. Filters were treated as described and cells were counted. Graphs represent the average of triplicate values for each experiment. Error bars shown represent standard deviations.

### 4.7 Xenograft assay

Nu/J (Nude; Jackson Labs, Bar Harbor, ME, USA) or NOD.CB17‐*Prkdc* ^*scid*^ */*NcrCrl (Scid; Charles River Labs, Raleigh, NC, USA) mice were used for the tumorigenicity studies. Mice were injected with 1 × 10^7^ cells subcutaneously on the flanks and tumors allowed to grow to a maximum size of 1 cm^3^ (Vaughan *et al*., 2012b). For the xenograft assays where transfections were carried out prior to injection, we counted the cells after transfection at the day of injection (48–72 h post‐transfection). Graphs represent the average of triplicate values for each experiment. Error bars shown represent standard deviations.

### 4.8 Western blotting

Immunoblotting was performed as described (Scian *et al*., 2005). Following Abs were used: p53 antibody pAb 1801 (Banks *et al*., 1986). Notch1 (3608S), Notch2 (4530P), Notch3 (5276P), Notch4 (2423S), IGF1R (9750S), HELLS (7998S), PIM1 (3247S), and TGFβ (3711S) were from Cell Signaling (Beverly, MA, USA). CCNB2 (sc‐5238), CREB (sc‐186), Ets‐1 (sc‐350), E2F1 (sc‐22820), Mcl1 (sc‐819), and Sp1 (sc‐17824) antibodies were from Santa Cruz Biotechnology (Dallas, TX, USA). Axl Ab (H0000558‐M01) was from Abnova (Taipei City, Taiwan), NFκB2 Ab (05‐361) was from Millipore (Billerica, MA, USA), and Tubulin Ab (T5326) was from Sigma Aldrich (St. Louis, MO, USA). Westerns blots were developed by the ECL method (GE Healthcare, Piscataway, NJ, USA).

### 4.9 Chromatin immunoprecipitation

Chromatin immunoprecipitation assays were performed as described (Vaughan *et al*., 2013). p53 (DO1: sc‐126 and FL‐393: sc‐6243; Santa Cruz), acetylated histone H3 (9K, 14K, 17‐615; Millipore), and IgG (normal mouse: sc‐2025 and normal rabbit: sc‐2027; Santa Cruz) antibodies were used for ChIP. QPCR was used to quantify precipitated DNA using promoter‐specific primers. Graphs represent the average of triplicate values for each experiment. Error bars shown represent standard deviations. The primer sequences are given in Table S11.

### 4.10 RNA sequencing and ChIP sequencing

Total RNA was prepared using the Trizol reagent from Invitrogen. Total RNA was sent to the Donnelly Sequencing Centre (University of Toronto, Canada) that carried out the library preparation and sequencing. mRNA libraries were generated from 4 μg of total RNA using Illumina TruSeq RNA Sample Prep V2 kits (RS‐122‐2001) per the manufacturer's directions.

ChIP‐seq for p53 and acetylated H3 histone (acetylated at 9K and 14K) using H1299 cells expressing p53‐R273H (or vector control) was performed as described previously (Vaughan *et al*., 2013). ChIP‐seq libraries were generated from 20 ng of input material using NEB Next ChIP‐seq Library Prep kit (E6240S/L) and NEB Next Oligos (E7500L). The manufacturer's protocol was followed; adapter‐ligated and PCR‐amplified DNA was purified using Ampure XP beads (A63881, Agencourt, Beckman Coulter, Indianapolis, IN, USA) and multiplexed prior to 2% agarose gel purification achieving a median size distribution of 311 bp. Sequencing was completed on the Illumina HiSeq2500 platform using version 3 chemistry and reagents. Single read data (50 bp) were processed with RTA 1.17.21.3, HCS 2.0.10.0 and aligned with casava V1.8.2 secondary analysis package (Illumina, San Diego, CA, USA). Four to ten million reads were analyzed by Illumina HiSeq, and the data were uploaded as fastq files that were analyzed by the DNASTAR program ArrayStar using the peak detection method QSeq Peak Finder. The QSeq Peak Finder is based on the ERANGE 3.1 Algorithm for ChIP‐seq and RNA‐seq analyses (Mortazavi *et al*., 2008). This peak detection algorithm calculates peaks in a normalized reads‐per‐million space. This algorithm also considers the directionality of reads when calling peaks. The ChIP‐seq Peak Finder reports the number of reads within the peak as the score for that peak. In addition, the ChIP‐seq Peak Finder reports a *P*‐value that contains the likelihood of a given region being a ‘true’ peak. The *P*‐value is based on how many peaks QSeq would have called in the control lane. The minimum number of hits in a particular genomic location was set to 4 and the minimum fold enrichment over control was set to 4. The minimum fold enrichment value specifies how many more reads must be in a region versus the same region in the control lane to be considered a peak. The analysis flow‐through allows users to designate certain files as controls. H1299 cells stably expressing p53‐R273H which were ChIP'd with IgG and then sequenced were designated as the control for the p53 ChIP sample. RNA‐seq accession #: ArrayExpress accession E‐MTAB‐5652. ChIP‐seq accession #: ArrayExpress accession E‐MTAB‐5653.

### 4.11 Differential gene expression analysis between GOF p53 tumor samples and wild‐type and null p53 tumors

RNA‐seqV2 data set for LUADS was downloaded from TCGA data portal (note that this portal is no longer operational and data are now available from the Genomic Data Commons). Data from rsem.genes.normalized_results files were used unmodified for gene expression quantitation. The p53 mutation status for each tumor sample was determined from TCGA's Curated Somatic Mutations file. Tumor samples with hotspot GOF p53 mutations were identified. Hotspot mutations were at codons 175, 245, 249, 273, 280, and 281. Samples without GOF p53 mutations, either wild‐type or null p53 mutations, were identified and grouped. Statistical analysis for gene expression differences between the GOF p53 sample population and the wild‐type/null p53 mutation sample population was based on the *t*‐test with the Benjamini–Hochberg correction for multiplicity and a false discovery rate of 0.05 (*P*‐values were uncorrected).

### 4.12 ChIP‐Re‐ChIP assays

ChIP‐re‐ChIP was performed following the method described (Furlan‐Magaril *et al*., 2009) by incubating equal amounts of extracts with p53 and IgG antibodies overnight and then incubating with BSA and sonicated salmon sperm‐saturated protein A agarose beads for 1 h at 4 °C. The DNA/protein/antibody complexes were then washed once with RIPA (150 mm NaCl, 50 mm Tris pH8, 0.1% SDS, 0.5% sodium deoxycholate, 1% NP‐40), once with high salt buffer (500 mm NaCl, 50 mm Tris pH 8, 0.1% SDS, 1% NP‐40), once with LiCl buffer (250 mm LiCl, 50 mm Tris pH 8, 0.5% sodium deoxycholate, 1% NP‐40), and once with 1× TE. DNA/protein complexes were eluted from the protein A agarose beads by incubation at 37 °C for 30 min in 10 mm DTT in 1× TE. Eluents were then incubated with the indicated secondary antibody overnight, and BSA and sonicated salmon sperm‐saturated protein A agarose beads were added for 1 h at 4 °C the following day. The DNA/protein/antibody complexes were then washed once with RIPA, once with high salt buffer, once with LiCl buffer, and once with 1× TE. Regular ChIP procedure was performed after that. Graphs represent the average of triplicate values for each experiment. Error bars shown represent standard deviations.

### 4.13 Chromatin opening assay

Samples were prepared for ChIP as described above, immunoprecipitated, and washed. DNA/protein complexes bound to protein A agarose were incubated with specific restriction enzymes, the DNA were purified, and QPCR was performed with specific primers on either side of the ‘loop’. Prospective restriction enzymes were identified using NEB Cutter software. Primers used were the same as those used for ChIP and are listed in Table S12.

### 4.14 Sphere forming assay

Cells were plated in ultra‐low attachment plates (Sigma) at a cell density of 5E4 cells/well of a 24‐well plate. The base medium used was Dulbecco's modified Eagle's medium : F12 supplemented with 4 μg·mL^−1^ insulin, 20 ng·mL^−1^ epidermal growth factor (EGF), 20 ng·mL^−1^ bFGF, and B27 at a final concentration of 2% as described (Eramo *et al*., 2008; Justilien *et al*., 2012). Pictures were taken three to 4 days after plating.

### 4.15 Statistical analysis

All statistical analyses were calculated using an unpaired, two‐tailed, equal variance Student's *t*‐test. Where multiple data sets were compared with each other, an ANOVA was calculated. FDR Benjamini–Hochberg multiple testing correction was implemented and data were considered significant if the *P*‐value was below 0.05.

## Author contributions

CV and SS performed all experiments; CV generated and analyzed ChIP‐seq and RNA‐seq data; BW analyzed TCGA data; SRG and SPD contributed to experimental design; and SD designed the experiments, supervised the project, and wrote the manuscript with additional editing by CV, SS, SRG, and SPD.

## Supporting information


**Fig. S1.** ChIP‐sequencing using lung cancer cells expressing GOF‐mutant p53‐R273H.

**Fig. S2.** Verification that GOF p53 does not interact on regions of DNA with no binding as shown through ChIP‐seq analysis.

**Fig. S3.** Verification that GOF p53 does not interact on the regulatory sequences of indirect target genes.

**Fig. S4.** qPCR showing upregulation of mutant p53 target genes.

**Fig. S5.** GOF p53 induces tumor‐initiating cell (TIC)‐related genes and the Notch pathway.

**Fig. S6.** Functional importance of multiple oncogenic pathways in GOF activities of mutant p53.

**Fig. S7.** Parallel oncogenic pathways activated by GOF p53.

**Fig. S8.** ChIP‐seq showing acetylated histone H3 and p53 peaks at GOF p53 target genes.

**Fig. S9.** Verification that siRNA reduces transcription factor expression.

**Table S1.** Complete list of genes that have p53 bound to the promoter.
**Table S2.** The complete list of genes that are up‐regulated by p53‐R273H at least 3‐fold in comparison to control (stably transfected with vector alone).
**Table S3.** The complete list of genes that are up‐regulated by p53‐R273H in comparison to control and are bound by 273H = DIRECT targets.
**Table S4.** TCGA Table.
**Table S5.** Identification of several example genes considered to be indirect targets of mutant p53.
**Table S6.** The complete list of genes that are up‐regulated by p53‐R273H in comparison to control and are not bound by 273H = INDIRECT targets.
**Table S7.** Functional importance of genes found in each functional category from Table 1.
**Table S8.** Complete list of genes that have AcH3 bound to the promoter.
**Table S9.** Direct targets of p53‐R273H, which have increased AcH3 histone on chromatin on their regulatory sequences, in H1299 cells expressing p53‐R273H compared to empty vector.
**Table S10.** Genes in common between p53 and AcH3 ChIP‐seq.
**Table S11.** Requirements of TFs for three direct target genes.
**Table S12.** Primer sequences used.
