# Supplementary material for: Gain‐of‐function p53 activates multiple signaling pathways to induce oncogenicity in lung cancer cells
Source: Mol Oncol. 2017 May 8;11(6):696–711. doi: 10.1002/1878-0261.12068 (PMC5467493; doi:10.1002/1878-0261.12068)

A. Western analysis of endogenous p53 levels.

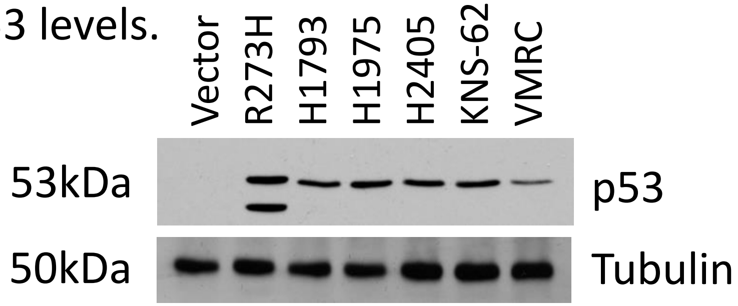

B. Immunoprecipitation of p53 from different lung cancer cell lines.

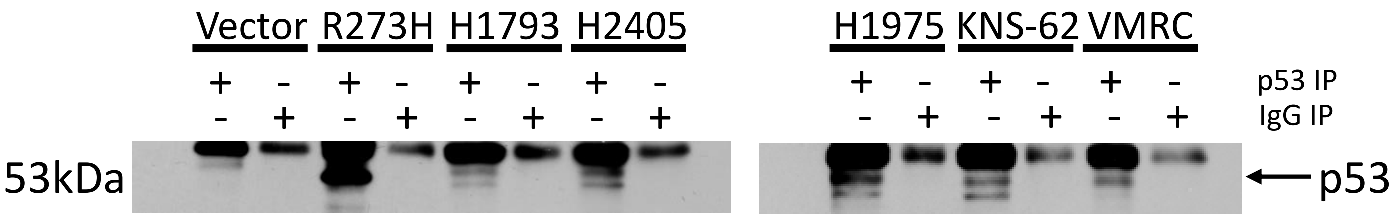

C. Chromosome 12

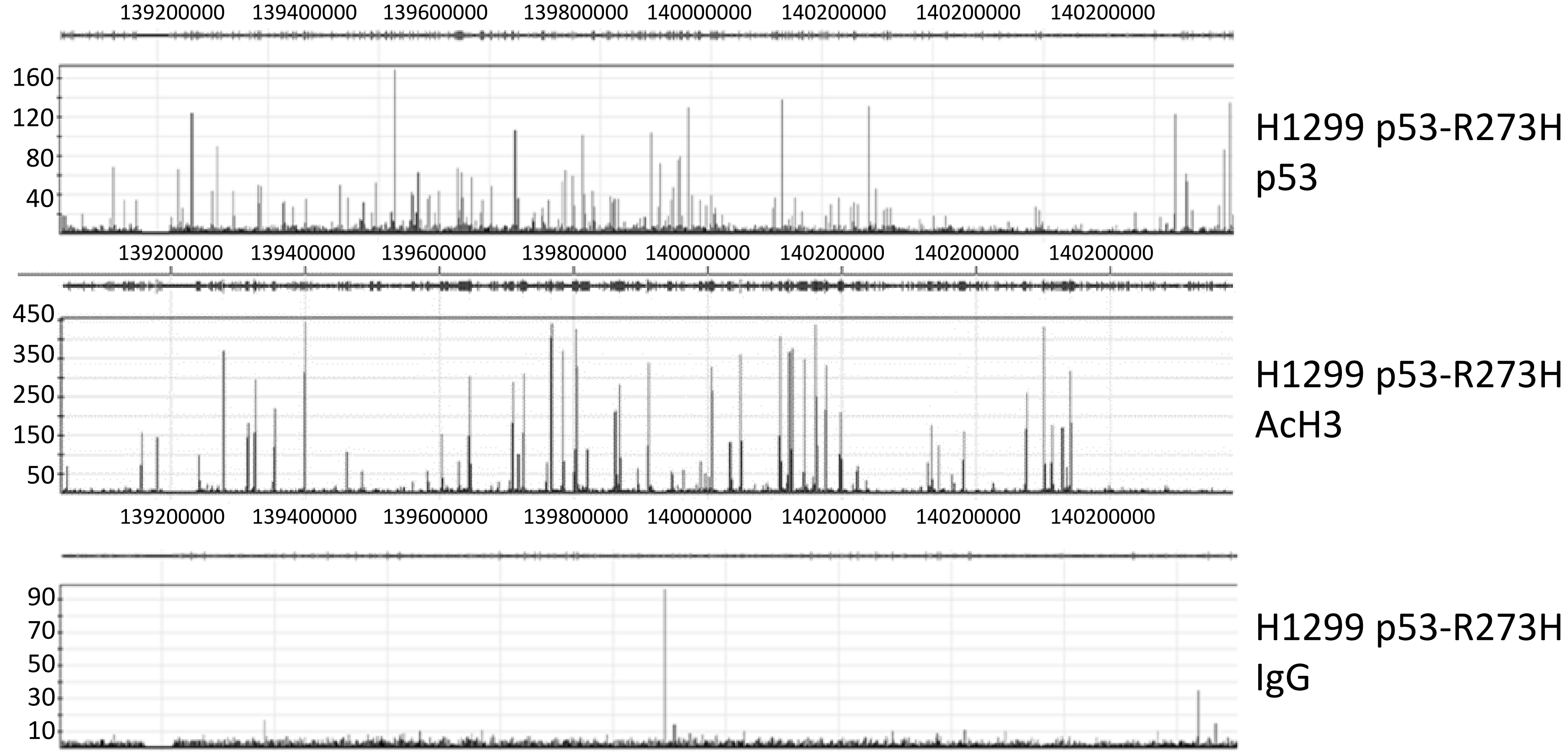

Supplement: Supplementary file 1 — Fig. S1. ChIP‐sequencing using lung cancer cells expressing GOF‐mutant p53‐R273H. [file MOL2-11-696-s001.pdf]
