# Supplementary material for: Gain‐of‐function p53 activates multiple signaling pathways to induce oncogenicity in lung cancer cells
Source: Mol Oncol. 2017 May 8;11(6):696–711. doi: 10.1002/1878-0261.12068 (PMC5467493; doi:10.1002/1878-0261.12068)

A. QPCR evaluation of p53 binding to control regions of DNA

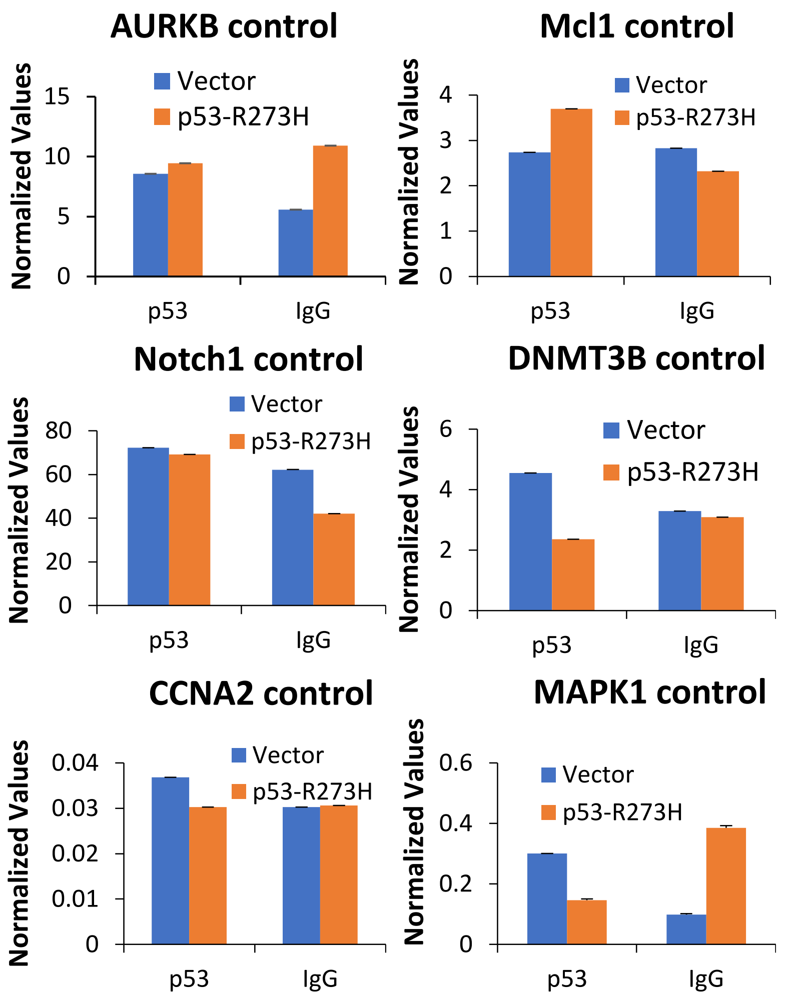

B. QPCR evaluation of AcH3 binding to control regions of DNA

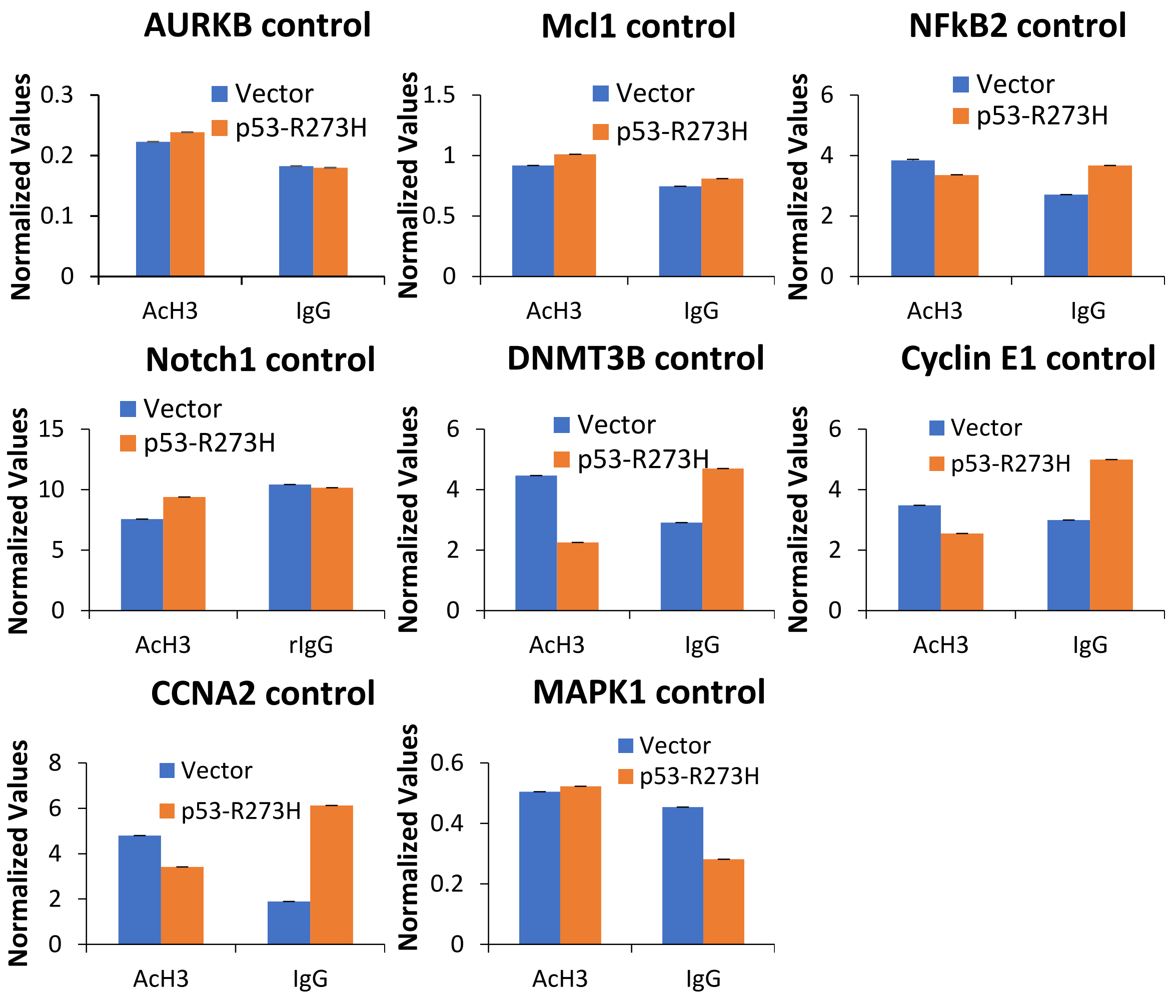

Supplement: Supplementary file 2 — Fig. S2. Verification that GOF p53 does not interact on regions of DNA with no binding as shown through ChIP‐seq analysis. [file MOL2-11-696-s002.pdf]
