# Supplementary figures and images for: Gain‐of‐function p53 activates multiple signaling pathways to induce oncogenicity in lung cancer cells
Source: Mol Oncol. 2017 May 8;11(6):696–711. doi: 10.1002/1878-0261.12068 (PMC5467493; doi:10.1002/1878-0261.12068)

A. QPCR evaluation of ChIP indirect targets

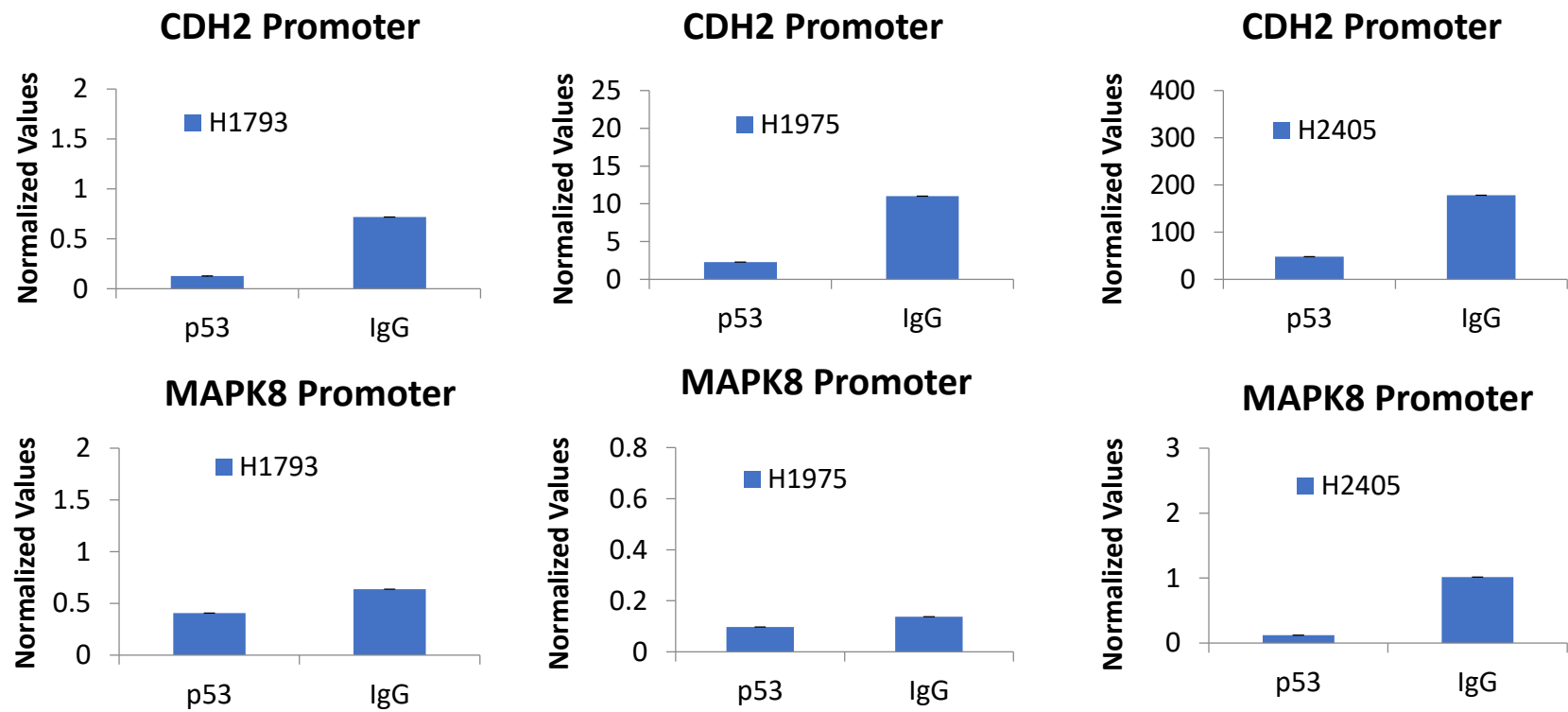

Supplement: Supplementary file 3 — Fig. S3. Verification that GOF p53 does not interact on the regulatory sequences of indirect target genes. [file MOL2-11-696-s003.pdf]

A.

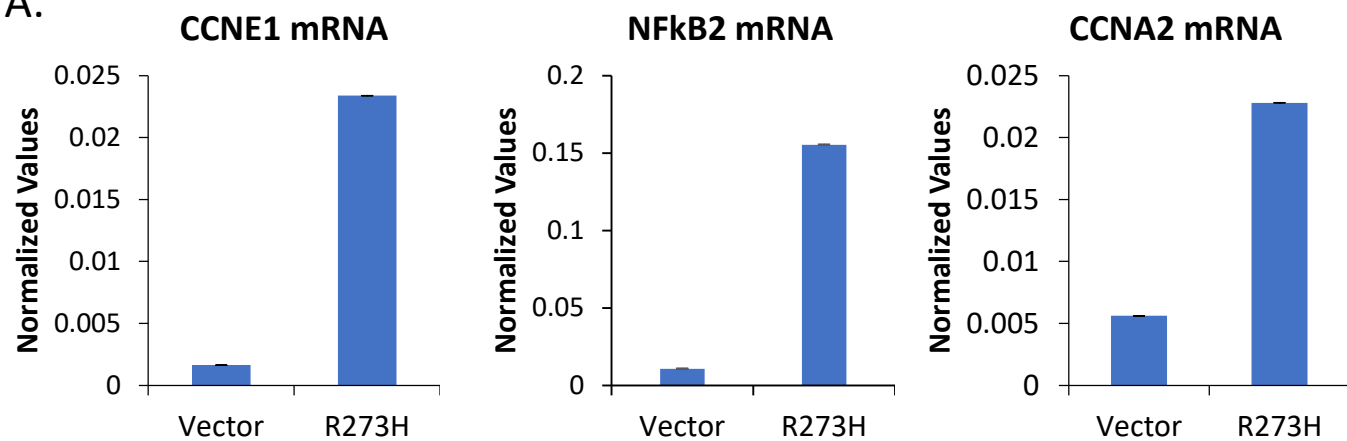

B.

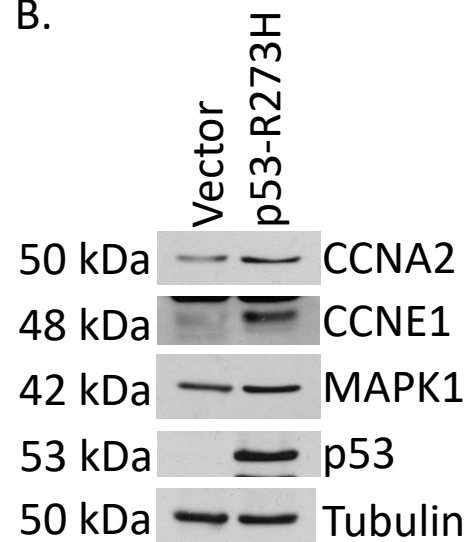

Supplement: Supplementary file 4 — Fig. S4. qPCR showing upregulation of mutant p53 target genes. [file MOL2-11-696-s004.pdf]

A. Fold increase in TIC gene expression in H1299 expressing p53-R273H

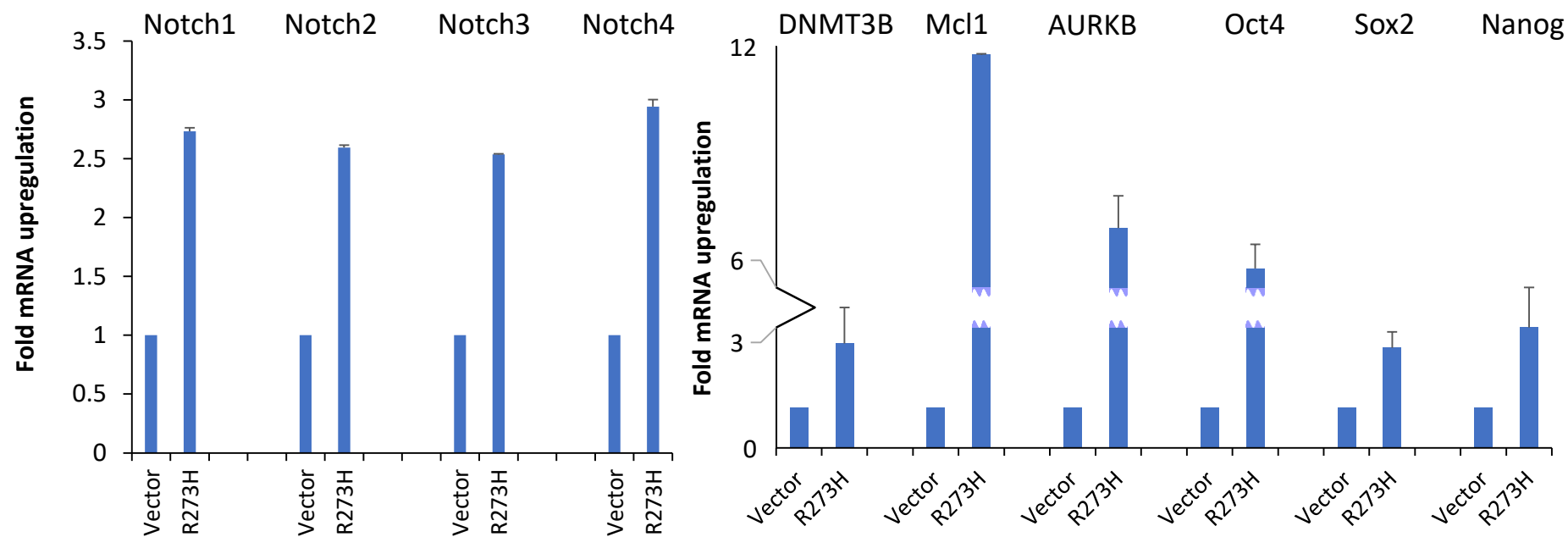

B.

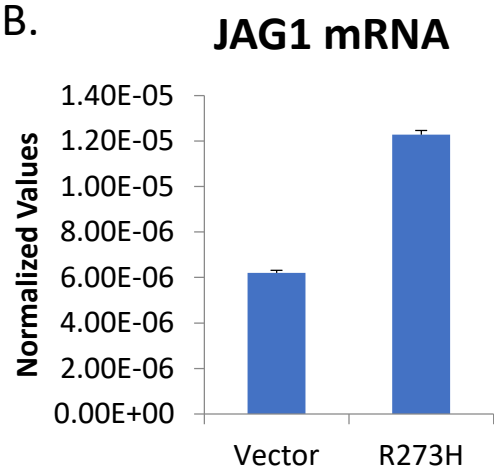

Supplement: Supplementary file 5 — Fig. S5. GOF p53 induces tumor‐initiating cell (TIC)‐related genes and the Notch pathway. [file MOL2-11-696-s005.pdf]

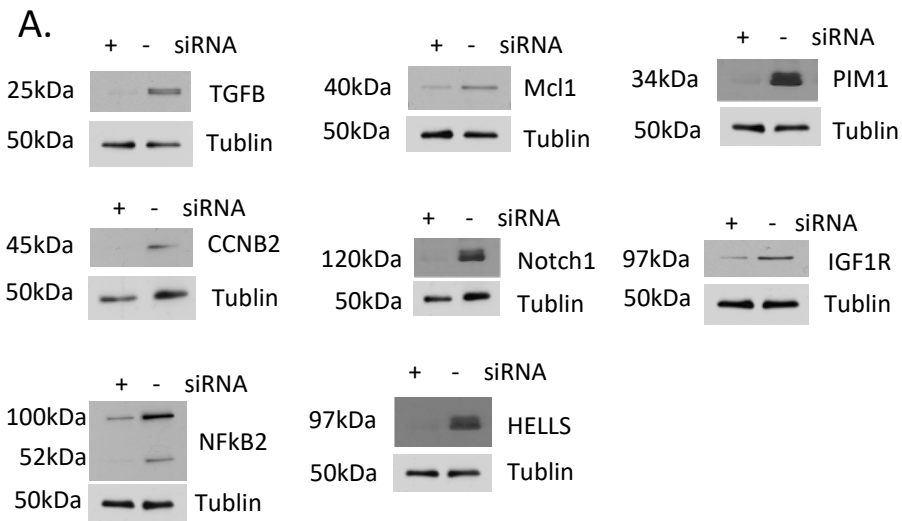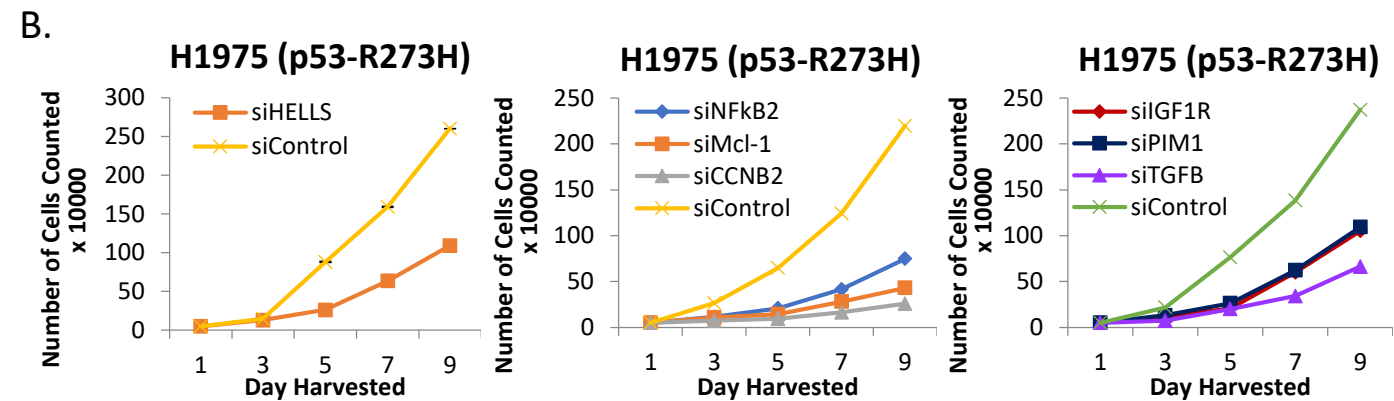

**C.** H1975 (p53-R273H) Migration Assay

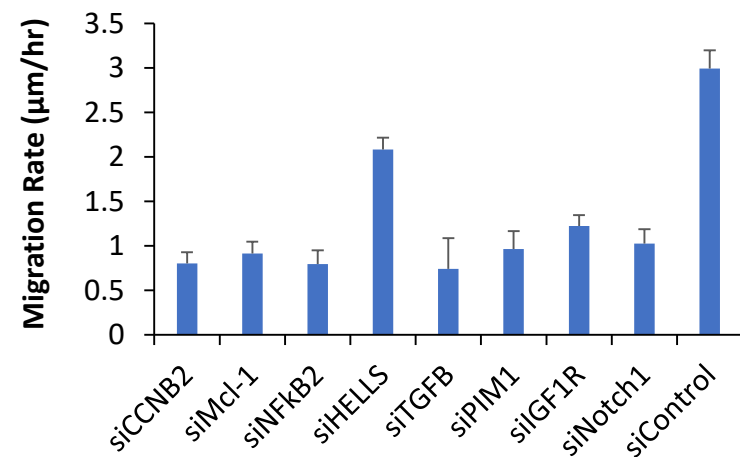

H1975 (p53-R273H) Invasion Assay

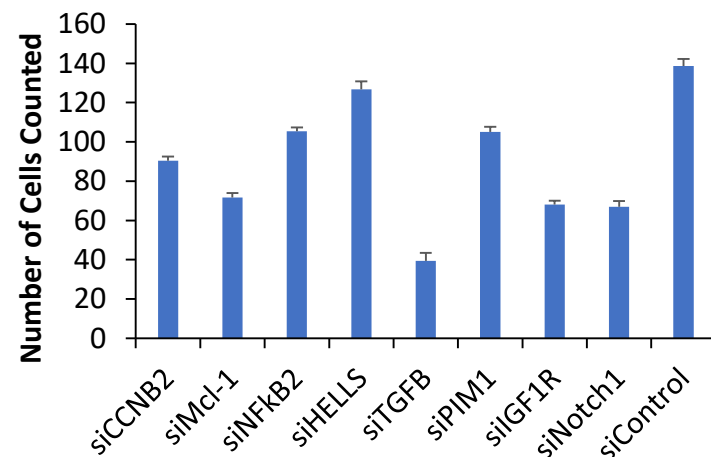

**D.** Sphere forming assay after knockdown with siRNA.

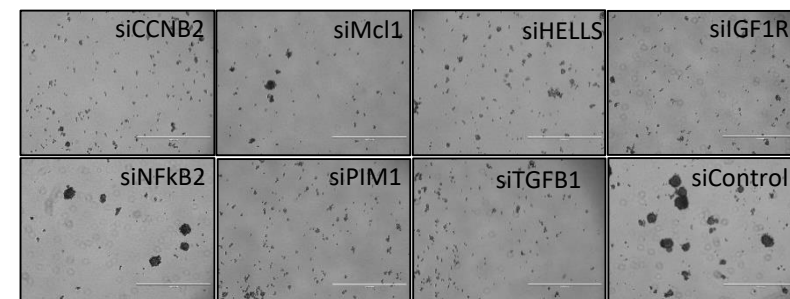

Supplement: Supplementary file 6 — Fig. S6. Functional importance of multiple oncogenic pathways in GOF activities of mutant p53. [file MOL2-11-696-s006.pdf]

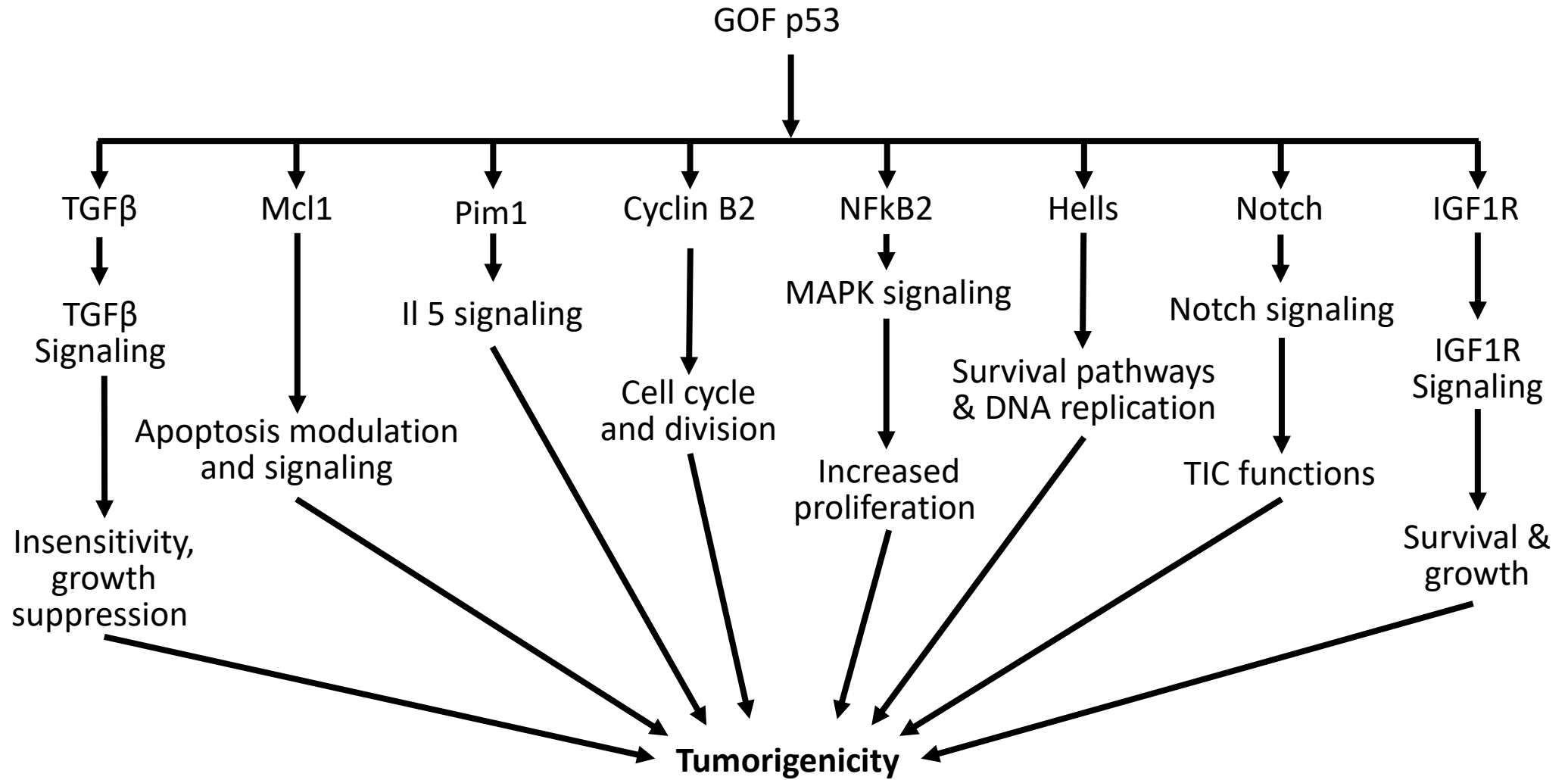

Supplement: Supplementary file 7 — Fig. S7. Parallel oncogenic pathways activated by GOF p53. [file MOL2-11-696-s007.pdf]

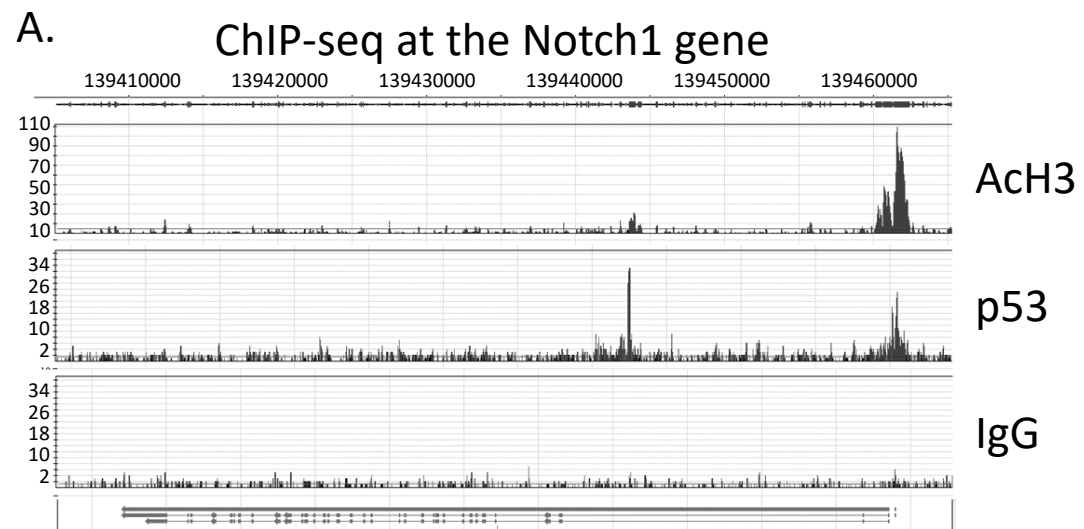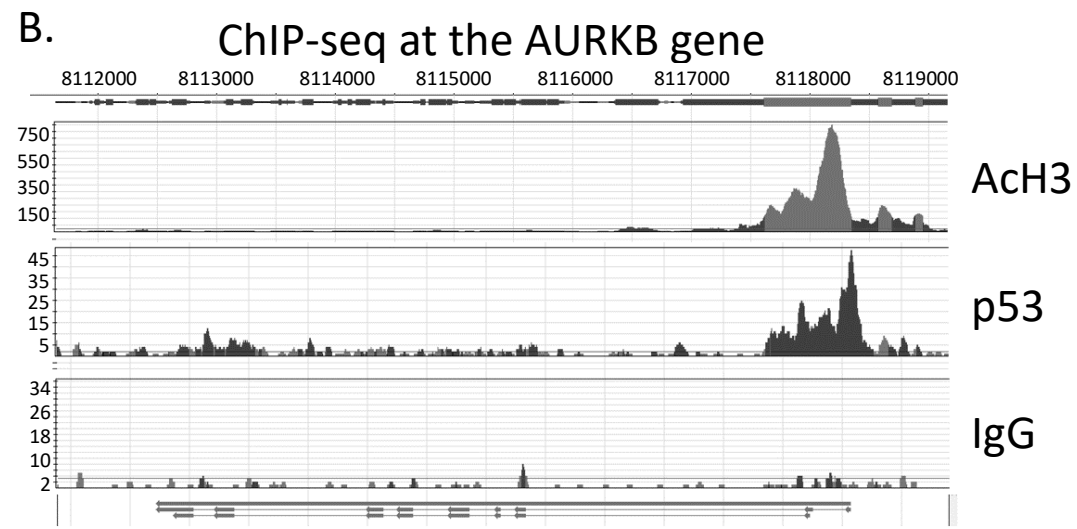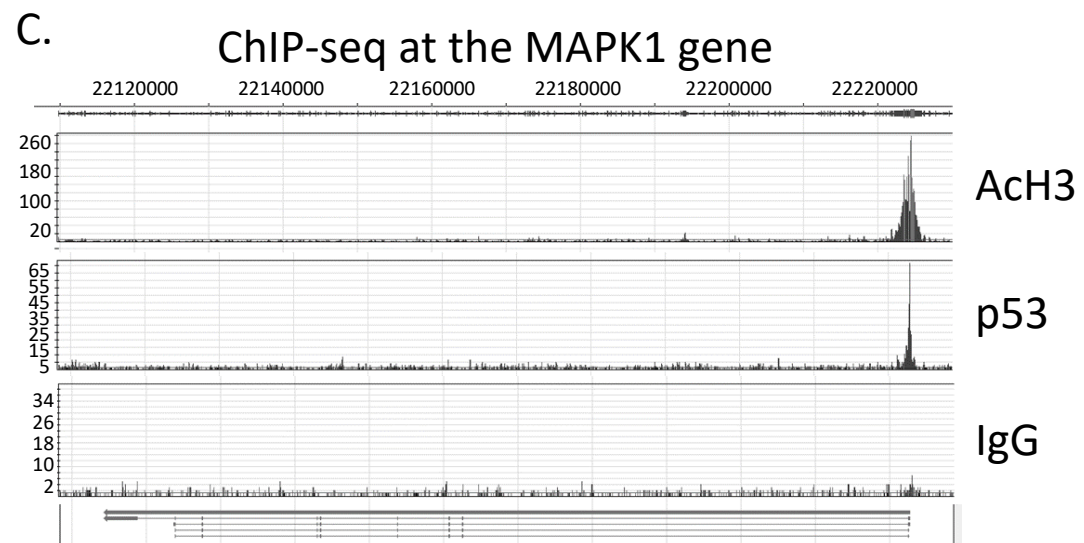

Supplement: Supplementary file 8 — Fig. S8. ChIP‐seq showing acetylated histone H3 and p53 peaks at GOF p53 target genes. [file MOL2-11-696-s008.pdf]

# A. Western analysis of siRNA against transcription factors

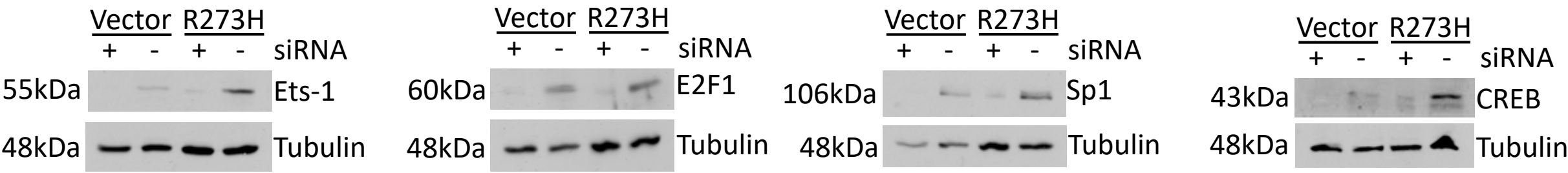

## B.

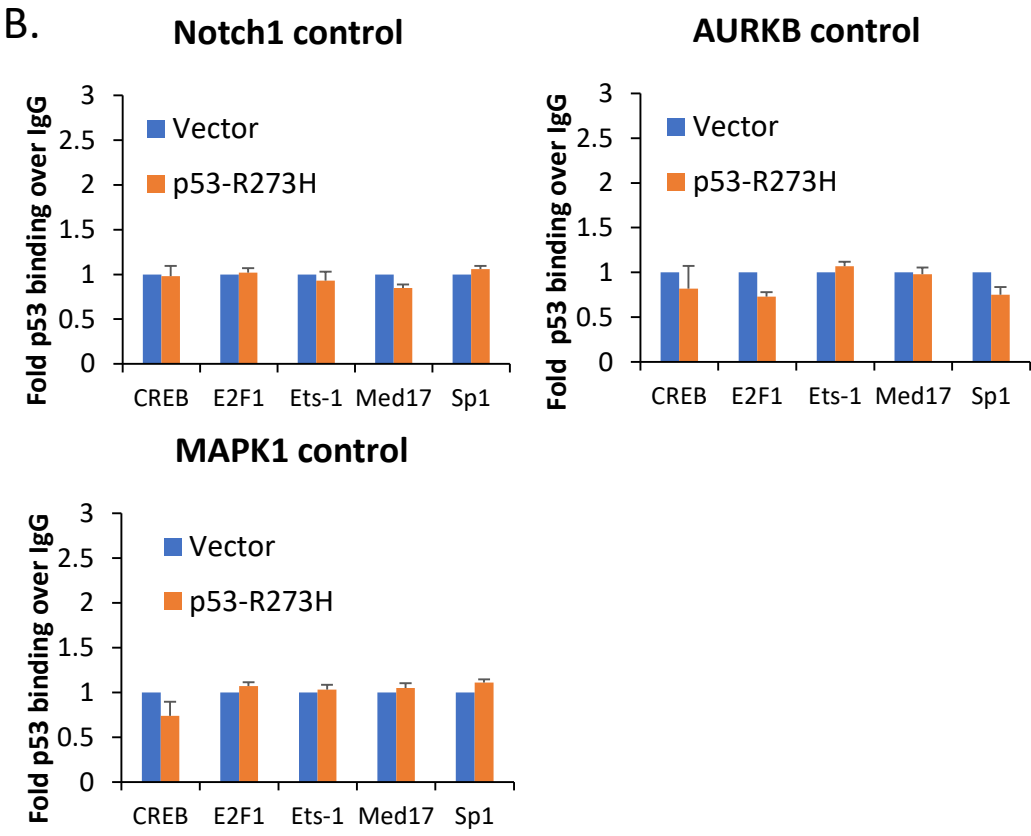

## C.

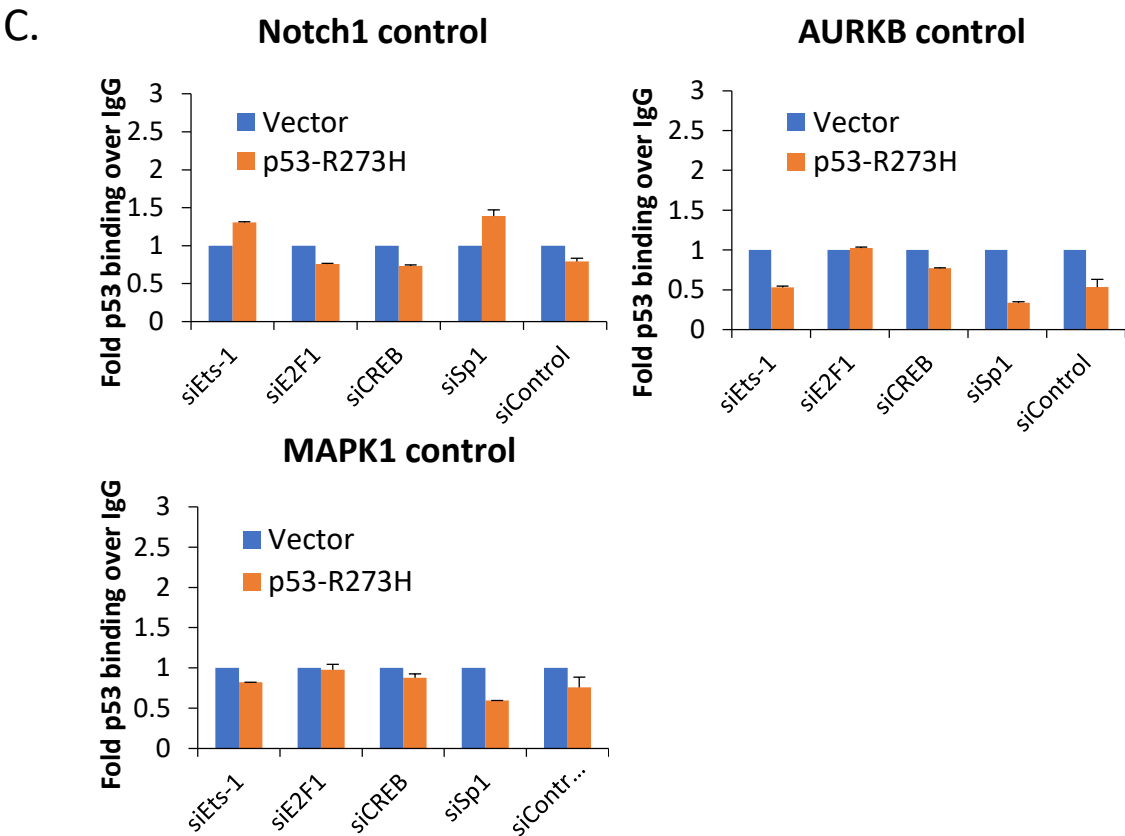

Supplement: Supplementary file 9 — Fig. S9. Verification that siRNA reduces transcription factor expression. [file MOL2-11-696-s009.pdf]
